# Trampoline-Associated Cranial and Spinal Injuries: A 10-Year Study in a Pediatric Neurosurgery Center

**DOI:** 10.7759/cureus.39097

**Published:** 2023-05-16

**Authors:** Joe M Das, Azam Baig, Nyararai Togarepi, Wai Cheong Soon, Pasquale Gallo, A Richard Walsh, Guirish A Solanki, Desiderio Rodrigues, William B Lo

**Affiliations:** 1 Pediatric Neurosurgery, Birmingham Children's Hospital, Birmingham, GBR; 2 Spine Surgery, Salford Royal NHS Foundation Trust, Salford, GBR

**Keywords:** pediatrics, neurosurgery, spinal injury, cranial injury, head injury, trampoline

## Abstract

Objective: There has been an increasing use of trampolines for recreation by children in recent years. Many studies have explored the different types of injuries sustained due to falls from trampolines, but so far none have focused specifically on cranial and spinal injuries. In this study, we describe the pattern of cranial and spinal injuries sustained by pediatric patients that were associated with the use of trampolines and their management in a tertiary pediatric neurosurgery unit over a period of 10 years.

Methods: This is a retrospective study of all children less than 16 years of age with suspected or confirmed trampoline-associated cranial or spinal injuries, managed by a tertiary pediatric neurosurgery unit from 2010 to 2020. Data collected included the patient's age at the time of injury, gender, neurological deficits, radiological findings, management, and clinical outcome. The data were analyzed to highlight any trends in the pattern of injuries.

Results: A total of 44 patients with a mean age of 8 years (ranging from one year and five months to 15 years and five months) were identified. 52% patients were male. 10 patients (23%) had a reduced Glasgow Coma Scale (GCS) score. In terms of imaging findings, 19 patients (43%) had a radiologically positive head injury, nine (20%) had a craniovertebral junction (CVJ) injury, including the first (C1) and second (C2) cervical vertebrae, and six (14%) had an injury involving other parts of the spine. No patient sustained concurrent head and spinal injuries. Eight (18%) patients had normal radiological findings. Two (5%) had incidental findings on radiology that required subsequent surgery. A total of 31 patients (70%) were managed conservatively. 11 patients (25%) underwent surgery for their trauma, of which seven were cranial. Two further patients underwent surgery for their incidental intracranial diagnoses. One child died from an acute subdural hemorrhage.

Conclusions: This study is the first to focus on trampoline-associated neurosurgical trauma and report the pattern and severity of cranial and spinal injuries. Younger children (less than five years of age) are more likely to develop a head injury, whereas older children (more than 11 years of age) are more likely to develop a spinal injury following the use of a trampoline. Although uncommon, some injuries are severe and require surgical intervention. Therefore, trampolines should be used prudently with the appropriate safety precautions and measures.

## Introduction

The history of the trampoline began in 1945, when it was first registered as a tumbling device by George Nissen, a competitive gymnast [1]. It was used for training acrobats, gymnasts, and military aviators. However, the potential for recreational use was noted, and soon trampolines were available on the market for indoor and home leisure. The current coronavirus pandemic saw an increase in injuries related to trampoline use in the United Kingdom (UK), due to more time spent at home and limited access to sports and outdoor activities [2-4]. Conventional trampolines use coiled springs to generate a bounce of height when children jump on them. However, improper use, including inappropriate installation, overcrowding by multiple children, or a lack of parental supervision during play, can result in injuries upon landing. Trampoline use has been described as being associated with many injuries, predominantly in the extremities, followed by head and cervical spine [1]. The American and Canadian academies of pediatrics, orthopedics, and sports have discouraged the recreational use of trampolines in general [5]. Thus far, there has been no neurosurgical study on trampoline-related injuries and their outcomes. Here we describe the pattern of trampoline-related cranial and spinal injuries in children who were referred to a tertiary neurosurgery unit, which is part of a regional major trauma hospital.

## Materials and methods

Children with both suspected and confirmed cranial and/or spinal injuries from 2010 to 2020 were identified from the prospectively maintained departmental electronic referral database. Information on age, sex, neurological examination findings, associated injuries, radiological imaging findings, management, follow-up, and clinical outcomes was extracted from the referral database, imaging system, and operative and medical notes. Ethical approval was not required for this retrospective, non-interventional study.

The patients were grouped according to their ages: 1-5, 6-10, and 11-16 years. The cut-off age of six years was used because the Royal Society for the Prevention of Accidents (RoSPA) recommended that trampolines are not suitable for children under the age of six [6].

Descriptive statistics were calculated as percentages, and the correlation among variables was tested. Statistical Product and Service Solutions (SPSS) 20.0 was used for statistical analysis. A p-value of less than 0.05 was considered significant.

## Results

A total of 44 patients, all under the age of 16, were identified. 23 (52%) of the patients were male and 21 (48%) were female. The mean age was eight years (range: one year five months-15 years five months). 14 children were 1-5 years old, 17 were 6-10 years old, and 13 were teenagers (11-16 years).

Mechanisms of injury

28 patients sustained falls. Two injuries were related to children playing together on the trampoline, although it was not clear whether the injuries were from collisions between children or from falls. The rest, 14 patients, had different mechanisms, including hitting on the steel rim. Equipment malfunctions or failures were not reported in any of the cases.

Presenting clinical features and associated injuries

28 (64%) children were symptomatic upon presentation to the hospital. 10 (23%) patients had reduced consciousness levels (Glasgow Coma Scale (GCS) score 3-14) at some point; the other clinical features were neck pain, paraesthesia in the limbs, torticollis, and weakness of the extremities in descending order of frequency (Table 1). 16 (36% of the children) were asymptomatic and had a GCS score of 15. Only one of the 44 children had an associated non-neurosurgery injury, namely a right clavicular fracture, along with the primary craniovertebral junction (CVJ) injury resulting from the trampolining accident. 

**Table 1:** Presenting features of patients after sustaining injuries from trampoline GCS: Glasgow Coma Scale

Clinical features	N	%
Reduced GCS score	10	23
Neck pain	6	14
Paresthesia	5	11
Torticollis	4	9
Limb weakness	3	7
None	16	36

Radiological findings and anatomical sites of injury

Based on the radiological findings, the children were grouped according to the specific sites of the injury: cranial, CVJ, spine excluding CVJ (i.e., subaxial cervical, thoracic, and lumbar spine), incidental finding, and no injury seen (Table 2).

**Table 2:** The demographic, clinical, and radiological data for the different age groups CVJ: Craniovertebral junction

		N	%	Age groups (years)		
				1 to 5	6 to 10	11 to 16
Number				14	17	13
Sex	Male	23	52	8	11	4
	Female	21	48	6	6	9
Neurological deficit	Yes	28	64	7	11	10
	No	16	36	7	6	3
Associated injuries	Yes	1	2	1	0	0
	No	43	98	13	17	13
Imaging	Cranial injury	19	43	10	8	1
	CVJ	9	20	1	5	3
	Spine	6	14	0	2	4
	Incidental finding	2	5	1	0	1
	Normal	8	18	2	2	4
Treatment	Surgical	11	25	5	3	3
	Conservative	31	70	8	12	11
	Other	2	5	1	0	1

All children underwent CT scans as their primary radiological investigation. 19 (43%) CT scans showed a traumatic cranial abnormality, including 13 skull fractures (29.5%), three extradural hematomas (6.8%), and three subdural hematomas (6.8%). Nine (20%) patients had CVJ abnormalities confirmed on CT, including eight (18.8%) cases of rotatory subluxation and one (2.2%) second cervical vertebra (C2) spinous process fracture. There were six (14% of the total) cases of spinal injuries, excluding CVJ (Table 3).

**Table 3:** Pattern of injuries CVJ: Craniovertebral junction

Injury site	Injury type	N	%
Cranial	Parietal bone fracture	9	47
	Occipital bone fracture	2	11
	Frontal bone fracture	1	5
	Orbital wall fracture	1	5
	Extradural hemorrhage	3	16
	Subdural hemorrhage	3	16
CVJ	Rotatory subluxation	8	89
	C2 spinous process fracture	1	11
Spine	C5/6 anterolisthesis	1	17
	C6/7 subluxation and C7 wedge fracture	1	17
	Thoracic mild wedging	1	17
	Loss of lordosis	3	50

Two (5%) incidental findings were identified on the trauma CT scan. One of them showed a right cerebellar lesion, and another showed aqueductal stenosis with hydrocephalus. Eight (18%) CT scans showed no evidence of cranial or spinal injuries. The 1-5-year age group accounted for half of the cranial injuries (10/19), and the 11-16-year age group accounted for almost half of the spinal injuries (7/15), including those involving the CVJ (3/9). The trauma sub-analysis confirmed with the chi-square test that children under five years of age suffer a statistically significant greater frequency of head injuries, and children older than 11 years have a greater risk of spinal injuries.

Surgical and conservative management

Overall, 31 (70%) patients were managed conservatively, 11 (25%) patients underwent surgeries for their injuries, and two (5%) patients were treated for their incidental findings. The latter two were managed by posterior fossa craniotomy and excision of the cerebellar tumor, and an endoscopic third ventriculostomy for aqueductal stenosis. The falls that happened in these two patients might be due to ataxia secondary to their pathologies. We excluded these two patients from the analysis of trauma management.

Out of the 19 patients who sustained radiologically proven injuries to the head, 10 patients (53%) with skull injuries were conservatively managed. The nine (47%) cranial surgical procedures included craniotomies with evacuation of intracranial hematomas (three extradural, three subdural) and elevation of depressed skull fractures (3). All nine patients who had injuries to the CVJ were conservatively managed. Among the 6 patients with radiological injury to the rest of the spine, two (33%) were surgically managed, and four (67%) were conservatively managed. The two spinal procedures were posterior cervical fusion at C5-6 level for anterolisthesis and anterior cervical discectomy and fusion (C6-7 level) for C6-7 subluxation with a C7 wedge fracture. Five patients with CVJ injuries, two patients with spinal injuries, and two patients with normal imaging were managed conservatively with a collar. The other 22 patients were managed conservatively, including rest and analgesia.

The follow-up duration varied among patients (ranging from one week to two years). Follow-up data were unavailable for 13 patients (as most of them did not turn up for a follow-up), and the rest of the patients, except one, did not have any neurological deficits. One patient, who had sustained an atlanto-axial rotatory subluxation along with central cord edema extending from C4 to T1, and was conservatively managed, had residual right upper limb weakness at four months of follow-up. 

There was one death in our series. An 11-year-old boy, previously fit and well, suffered a direct head injury against the trampoline, which had multiple users at the time. One hour after sustaining the injury, he complained of headaches and became unresponsive. On the arrival of paramedics, the GCS was 3, which remained the same until he was intubated in the local emergency department, where his pupils were fixed and dilated. A CT head scan revealed a large right-sided subdural hematoma, subarachnoid hemorrhage, gross cerebral edema, midline shift, tonsillar herniation, and ‘reversal sign'. Within three hours, he was transferred to our unit, and he underwent an emergency decompressive craniectomy and evacuation of the subdural hematoma. Immediate post-operative intracranial pressure on the right side was 64 mm Hg. His intracranial hypertension persisted despite surgical decompression and maximal medical management in the intensive care unit. Sixteen hours later, brainstem death was confirmed, and sedation was discontinued. The boy died 12 hours later.

## Discussion

In the US, trampoline injuries doubled between 1991 and 1996 (from 39,000 to >83,000 per year). In 2009, the trampoline injury rates were 70/100,000 per year for children equal to or less than 5 years of age and 160/100,000 per year for children older than 5 years of age [5]. Other comparable activities with a similar injury profile were cycling and playground equipment use. Since children were exposed more to these activities than to trampolining, the number of injuries associated with them was higher [5,7,8].

The type of injury differed depending on the body part injured. Fractures or dislocations contributed to 64% of upper extremity injuries and 26% of lower extremity injuries, whereas the corresponding proportions for soft tissue injuries were 32% and 69%, respectively. Head and neck injuries (51%), including fractures or dislocations (1%), were uncommon. The approximate ratio of limb vs. head and neck vs. facial injuries was 7:1:1. The commonest facial injury was laceration (66%), followed by fractures or dislocations (9%) and dental trauma (3%) [9]. Our study added further data on the distribution of spinal injuries in addition to head and neck injuries.

Most of these injuries were treated in the emergency department; only 3.3% resulted in a hospital stay. The most serious injuries were to the head and neck [9]. These injuries have also resulted in deaths. Falls from the trampoline were the most frequent cause of death, followed by landing on the neck while attempting somersaults [5].

Trampolines are responsible for a significant number of injuries per year [5,10]. In a recent survey in the UK of 663 parents with a child under the age of 12, half of them reported that their child had hurt themselves on a trampoline [11]. Almost twice as many children are now sustaining fractures due to trampolines as they did a decade ago. It is clear the incidence is rising [12]. It is important to lay out the reasons for this and propose effective ways to reduce this problem.

An Australian study found that injuries from trampolines at commercial or sporting venues resulted in longer hospital stays, even though the number of trampoline injuries was higher (68.9%) at home [12]. Our data showed that in the pediatric population, head injuries resulting from trampolines were more common than CVJ injuries and spinal injuries. Cranial injuries were more common in the age group 1-5 years, and spinal injuries were more common in the age group 11-16 years, even though this was not statistically significant. This is likely due to the relatively greater head-to-body ratio in younger children. All the CVJ injuries showed neurological improvement with conservative management, whereas 47% of the cranial injuries transferred to our tertiary center required an operation. There was one patient mortality among our patients, a patient with a severe head injury and poor presenting GCS. In one New Zealand study, there were two mortalities over a 10-year period. Both were disabled, in residential care, and the events were unsupervised [13]. Findings from our contemporary study, including one case of mortality and one case of long-term neurological deficit, confirmed the potentially serious risk of trampolining.

According to the RoSPA, 60% of trampoline injuries occur when more than one person is using the trampoline at a time. It is, therefore, important to have one child at a time on the trampoline [14]. One common cause of injury was the children launching themselves off the trampoline. Such ‘bouncing exits’ can be prevented by keeping the safety netting closed while using most trampolines [15].

The American Academy of Orthopedic Surgeons states that children under the age of six should never use trampolines [16]. Safety guidelines were put forward by the RoSPA in the UK in 2015 [6]. It is advised that since children under the age of six are not sufficiently physically developed to control their bouncing and individuals weighing less are more likely to get injured, it is better to avoid trampolining in this age group. Other safety measures include taking turns with only one person using the trampoline at a time, avoiding somersaulting, having a trained ‘spotter’, avoiding combining alcohol with trampolining, and learning new skills from local trampoline clubs [6]. The latter two suggestions are less applicable to children.

The use of spring-free trampolines eliminates the hard-impact areas that can cause injury [17]. In windy weather, an anchoring kit will keep the trampoline in place, which is important. The surroundings should be padded with soft landing spots [18-20].

Various national, American, Australian, and international regulations and standards, including the RoSPA, the Consumer Product Safety Commission (CPSC), the Child Accident Prevention Foundation of Australia, and the International Association of Trampoline Parks, have been drawn up to ensure the standard and safety of trampolines, in terms of design, construction, and operation [6,16,21]. However, such measures would still not completely prevent equipment misuse and accidents, which are likely to be the main reasons for injuries.

In summary, trampoline-related injuries are on the rise. Clearly, the injuries can be severe but are rarely associated with mortality. While prevention is key, this contemporary study highlighted the pattern and severity of craniospinal injuries sustained associated with trampoline use in children, which is useful for both emergency department clinicians and neurosurgeons.

Limitations

In several cases, the exact mechanism of injury was not clear. For example, details such as the total number of children on the trampoline, which structure (or person) the child collided with, the height of the fall, the surface the child landed on, and the presence of a supervising adult are all related to the severity of the injury. Additionally, it was unclear whether the safety precautions recommended by guidelines and product manuals were adhered to. Moreover, no information was collected regarding the make of the trampoline, its height, or the safety measures available.

## Conclusions

We provide the first report on the pattern, management, and outcome of trampoline-associated cranial and spinal injuries in children. The use of trampolines can be associated with serious craniospinal injuries. Children under five years of age suffer a greater frequency of head injuries, and children older than 11 years of age have a greater risk of spinal injuries associated with the use of trampolines. By recognizing the potential injuries, emergency department clinicians and neurosurgeons can manage pediatric craniospinal injuries, associated with trampolines, which are on the rise.
